# Bridging the Gap: Third Places, Ageing, and Health in UK Roma Communities

**DOI:** 10.1093/geroni/igaf122.342

**Published:** 2025-12-31

**Authors:** Judith Sixsmith, Ryan Woolrych, Aleksandar Marinov, Margaret Greenfields

**Affiliations:** University of Dundee, Dundee, Scotland, United Kingdom; Heriot Watt University, Edinburgh, Scotland, United Kingdom; The Urban Institute, Heriot Watt University, Edinburgh, Scotland, United Kingdom; Anglia Ruskin University, School of Allied Health and Social Care, Cambridge, England, United Kingdom

## Abstract

Roma people, Europe’s largest ethnic minority, face systemic exclusion, discrimination, and health disparities, contributing to significantly lower life expectancy than the general population. In the UK, these inequities persist, particularly for older Roma individuals who navigate intersecting challenges related to health, housing, and social inclusion. The RomaPlaceAge Project, a research initiative co-led with Roma community members, explores the health and well-being experiences of Roma individuals aged 40+ across three case study sites - Govanhill (Scotland), Peterborough, and Luton (England). Central to this research is the role of third places - public and semi-public environments such as GP practices, outdoor spaces, community centres, and housing estates - where Roma individuals engage with services and social networks, often in the face of complex barriers including language difficulties, digital exclusion, and insecure housing. Through three knowledge café events with stakeholders (healthcare providers, NGOs, police, educators, and individuals with lived experience), and in-depth interviews with Roma aged 40+, the project uncovers how third places either support or hinder health and well-being. Preliminary findings highlight the importance of micro-interactions and relationality in shaping access to services, the inaccessibility of healthcare services, the underutilisation of green spaces, and the role of community-led initiatives in fostering inclusion. By positioning third places as critical to health and well-being, this research contributes to discourses on participatory and place-based approaches to ageing, advocating for targeted interventions that promote equity, inclusion, and ageing well in the right place.

